# Model-based exploration of the rationality of off-label use of cetirizine in Chinese pediatric patients: a prospective cohort study

**DOI:** 10.3389/fphar.2024.1322788

**Published:** 2024-03-14

**Authors:** Wei Liu, Zhiyuan Tan, Ping Yang, Zhiheng Yu, Xueting Yao, Pengxiang Zhou, Ling Liu, Wei Zhou

**Affiliations:** ^1^ Department of Pharmacy, Peking University Third Hospital, Beijing, China; ^2^ Therapeutic Drug Monitoring and Clinical Toxicology Center of Peking University, Beijing, China; ^3^ Institute for Drug Evaluation, Peking University Health Science Center, Beijing, China; ^4^ Department of Obstetrics and Gynecology, Peking University Third Hospital, Beijing, China; ^5^ Drug Clinical Trial Center, Peking University Third Hospital, Beijing, China; ^6^ Department of Pediatrics, Peking University Third Hospital, Beijing, China

**Keywords:** cetirizine, allergic diseases, antihistamines, population pharmacokinetics, off-label use, modeling and simulation

## Abstract

**Aims:** Cetirizine is frequently administered at an increased dosage in clinical practice and recommended by several guidelines. Nonetheless, the pharmacokinetic (PK) profile and real-world safety data remain insufficient in the Chinese pediatric population. The objective of the current study is to develop a population pharmacokinetic (PPK) model for cetirizine in Chinese pediatric patients and to investigate the rationale behind its off-label usage.

**Methods:** A prospective cohort study was conducted, enrolling children who had been diagnosed with allergic diseases and prescribed cetirizine. The outcomes were safety and pharmacokinetic (PK) parameters. Cetirizine concentrations were measured using a pre-established analytical method. Subsequently, a PK model was developed, followed by model evaluation and simulation. The developed PK model was employed to investigate the drug exposure differences across various age groups and to simulate scenarios of potential overdose.

**Results:** Sixty-three children were enrolled, and 24 of them received a cetirizine dose exceeding the recommended dosage. A PPK model, based on published literature, served as the basis of our analysis, with adjustment made to estimate certain parameters. The final model evaluation and validation indicated accurate predictive performance and robust parameter estimation. Simulations conducted for the label-dose among age 1–12 indicated median maximum concentration at steady state (C_max,ss_) of 7 year old children could be the highest. The model was also used to predict the off-label dose scenarios and overdose patient to support the clinical decision. There were no adverse drug reactions in either group.

**Conclusion:** This study provides evidence-based and model-based exploration for optimizing cetirizine usage in Chinese pediatric patients. The cetirizine PPK model showed accurate predictive performance and could be utilized to simulate individual patient exposure in real-world clinical scenarios.

## 1 Introduction

Cetirizine is a second-generation H_1_ antihistamines (AHs) and is widely used for the treatment of allergic diseases, particularly in the treatment of allergic rhinitis and urticaria in children (Simons et al., 2000; Chen, 2008). Several domestic and international guidelines recommend escalating the dosage by 2–4 times when no clinical improvement is observed at the regular dose (Zuberbier et al., 2018; Caffarelli et al., 2019; Zhou et al., 2022). Nonetheless, such an escalation might lead to off-label usage of cetirizine, and it is important to note that these guideline recommendations are not uniformly consistent.

Off-label use of medications is a widespread practice worldwide, particularly in pediatric populations (Silva et al., 2014; Magalhães et al., 2015; Yackey et al., 2019). It is essential to establish a framework and evidence-based methodology that aids and supports clinicians in appropriately utilizing drugs not explicitly included in the medication instructions or labels authorized by regulatory agencies (Meng et al., 2022).

The guideline recommendations are primarily based on the clinical response and experience, however, a pharmacokinetic foundation was lacking to adequately support the rational utilization of this medication. For administering Cetirizine, the dosage is adjusted according to age, increasing from 2.5 mg once daily (qd) for children aged 6–11 months, to 5–10 mg qd for children older than 7 years. Cetirizine is predominantly eliminated by the kidneys, with its apparent clearance (CL/F) being age-dependent (Pitsiu et al., 2004). Given this characteristic, it is suggested that younger children receive lower yet more frequent doses. Nevertheless, the drug label recommends lower doses for younger ages without emphasizing the dosing frequency. In such circumstances, one might spectulate that when the clinical judgment calls for an increased dose, the appropriate dosing frequency may not always be utilized.

The issue arises from the recommended increased dosage, which is that the off-label use of the drug frequently occurs in clinical practice. Particularly, when calculating the dose based on weight (0.25 mg/kg) (Spicák et al., 1997; Pitsiu et al., 2004), it becomes easy to administer a dosage higher than the labeled dose for overweight and obese children.

Another issue deserving our attention is the safety profile of using an off-label drug, a profile that has not been fully assessed during pre-market clinical trials and, consequently, has not been rigorously assessed by regulatory agencies. This is where real-world research plays a critical role.

Although cetirizine’s long-term safety in treatment has been assessed in several studies, mainly concentrating on infants aged 12–24 months (Simons, 1999; Diepgen and Early Treatment of the Atopic Child Study Group, 2002; Simons et al., 2003), its pharmacokinetic (PK) profile and real-world safety data remain insufficient in Chinese pediatric patients. As of now, there exists no population pharmacokinetic (PPK) model specific to cetirizine usage in Chinese children, and thus, current drug dosing and adjustments are based on evidence derived from Western populations.

Therefore, this study undertook a real-world investigation of cetirizine in the Chinese pediatric population to elucidate the pharmacokinetic profiles of cetirizine in Chinese children and to assess the safety of escalated dosage across various age groups. These findings provide evidence-based reference for the rational administration of cetirizine in Chinese pediatric patients.

## 2 Materials and methods

### 2.1 Study design and inclusion/exclusion criteria

The data for this study originated from a prospective cohort study that received approval from the Ethics Committee of Peking University Third Hospital (No. 2020-227-02). Patient enrollment took place between April 2020 and April 2021. The inclusion criteria consisted of the following: 1) children aged under 14 who required oral cetirizine for managing allergic diseases, whether in an outpatient or inpatient setting, without regard to gender; 2) children with a clinical diagnosis of diverse types of allergies,

Exclusion criteria included the following: 1) abnormal liver function, with alanine aminotransferase (ALT) or aspartate aminotransferase (AST) levels that exceeded twice the upper limit of normal; 2) impaired kidney function, demonstrated by serum creatinine levels higher than the upper limit of normal or an estimated glomerular filtration rate (eGFR) less than 10 mL/min; 3) electrolyte abnormalities, specifically potassium or magnesium ions below 0.8 times the lower limit of normal, or above 1.2 times the upper limit of normal; 4) children with a history of severe adverse reactions to cetirizine; 5) children with severe allergies who required systemic or local administration of steroids.

Children underwent routine treatment with cetirizine as per clinical practice guidelines. If satisfactory disease control was not attained (e.g., in cases of uncontrolled asthma or urticaria, as determined by the asthma control test or urticaria activity score 7), the cetirizine dosage was escalated by 2–4 times the original dosage. Participants were then categorized into either a ‘label dose group’ if they remained within the recommended dosage or an ‘off-label dose group’ if they received more than the labeled dose. A maximum of three blood samples were taken from each participant for PK analysis, with each sample consisting of 3 mL of blood in an EDTA anticoagulated tube. After centrifugation at 3,000 rpm for 10 min, the plasma was preserved at −80°C. Informed consent was obtained from all participants.

### 2.2 Outcome indicators and information collection

Outcome indicators were classified into two categories: safety indicators and PK indicators. The safety indicators included liver and kidney functions such as SCr, urea, ALT, and AST. Pharmacokinetic data included the time of blood sampling, drug concentration levels, and the parameters and simulated data derive from the PK modeling. Patient demographics, disease-related information, and drug usage details, along with the outcome indicators, were documented through medical records. These primarily consisted of: 1) general information, including sex, age, weight, and height; 2) disease and treatment information, including diagnosis, duration of cetirizine therapy, dosage, administration frequency, and the time since the last dose prior to blood sampling; and 3) safety information, including SCr, urea, ALT, AST, WBC, and any unexpected adverse reactions that occurred during cetirizine treatment.

### 2.3 Blood sample analysis

The previously published HPLC-MS/MS method from our laboratory was utilized to determine the concentration of cetirizine in plasma samples obtained from children (Yang et al., 2021).

### 2.4 Data analysis

#### 2.4.1 Statistical analysis

Descriptive statistical analysis was conducted on the age, height, weight, and medication dosage information of the two groups of children. Count data were tabulated with frequency and composition ratio were expressed as percentages, whereas mean and standard deviation (x ± s) were computed for continuous data. For analyzing continuous variables, independent t-test were employed, and for categorical data, paired chi-square tests were utilized.

#### 2.4.2 Model analysis

Nonlinear Mixed-Effect Modeling software (NONMEM 7.4.4) compiled using gfortran ICON (United States) was employed for PPK modeling and simulation. Meanwhile, R 4.2.1 was utilized for data processing. Pachages such as xpose, xpose4, ggplot2, and others were harnessed to create diagnostic plots, statistical summaries of parameters, and model assessments.

The basic model was chosen due to the sparsity of the sampling strategy: a previously published cetirizine PK model in real-world Caucasian children [as reported by Pitsiu et al. (2004)] searved as the basis model for this study. The Pitsiu model described a one-compartment disposition process with linear absorption, where CL/F was linearly associated with age (CL_AGE) and gender (CL_SEX), and the apparent volume of distribution (V/F) was also linearly related to age (V_AGE). The absorption rate constant (Ka) was defined as the typical value of Ka plus CL/V, which depicted a flip-flop characteristic. The population typical values, inter-individual variability (IIV), and residual errors of all pharmacokinetic parameters were fixed according to the published model.

External evaluation of the basic model: Real-world data that was collected was utilized to conduct an external evaluation of the Pitsiu model. Individual PK parameters were derived through posterior Bayesian estimation. To assess the predictive performance of the published model on external datasets, goodness-of-fit (GOF) plots were generated, including scatter plots of dependent variables (DV) versus individual predictions (IPRED), DV versus population predictions (PRED), conditioned weighted residual erros (CWRES) plotted against PRED, and CWRES plotted against time after the last dose (TAD).

Model optimization: The typical values of CL_AGE, CL_SEX, V, V_AGE, IIV of CL, IIV of V, and residual errors were attempted to be estimated using the first-order conditional estimation with interaction (FOCE-I) method within the NONMEM software, to choose the final model for simulation.

Evaluation of the optimized model: The optimized model’s stability in terms of PK parameter estimation was evaluated using the bootstrap resampling method. Furthermore, we employed the normalized prediction distribution error (NPDE), based on 1,000 simulations, to assess the predictive accuracy of the optimized model.

Simulation of clinical pediatric off-label dosing and individualized dosing recommendations: The exposure metrics, including the maximum concentration at steady state (C_max,ss_) and the minimum concentration at steady state (C_min,ss_), as well as the PK curves over time for cetirizine in both labeled and off-label dose regimens, were simulated for children belonging to different age groups. Furthermore, posterior prediction (POSTHOC, MAXEVAL = 0 in NONMEM) was conducted for patients who received off-label doses or inadvertently suffered from serious overdose due to medication errors during clinical trials. These simulations were carried out to inform and support clinical decisions regarding dosing adjustments.

## 3 Results

### 3.1 Baseline characteristics

A total of 63 children were included in the study, with 38 males and 25 females. The average age was 6.34 ± 3.7 years, the average weight was 26.4 ± 15.7 kg, and their average height was 120.1 ± 27.7 cm. Of these children, 39 received regular doses as per the label instructions, and 24 received doses exceeding the recommended label dose. The distribution of children receiving off-label doses across various age groups and the corresponding ranges of actual doses administered are presented in Table 1.

**TABLE 1:** Number of patients in various age groups who received a dosage beyond the recommended range as stated on the label.

Age group	No.	Label dose	No. of patients exceeding label dose	Range of exceeding dose
0.5–2	10	2.5 mg bid	5	3–4 mg bid
2–6	22	2.5 mg bid	17	3.5–6.43 mg bid
6–12	33	5 mg bid	2	5.35–7.5 mg bid

Analysis of off-label drug usage revealed that, despite some children receiving doses higher than the recommended age-based dosage, only five children surpassed a dose of 0.25 mg/kg per administration (or 0.5 mg/kg/day), a higher dose used in the clinical trials (FDA, 2002a; FDA, 2002b). All administered doses fell within a two-fold range, with no children receiving more than double the recommended dose. The distribution of administered dosages based on body weight across various age groups is depicted in Supplementary Figure S1.

### 3.2 Model building and evaluation

Modeling was carried out by reproducing and evaluating Pitsiu model based on the PK samples included in the study. Firstly, an external evaluation was performed (Pitsiu et al., 2004), and it revealed misspecification across the entire exposure range, as depicted in Supplementary Figure S2. To address this, we took a step further by unfixing several parameters to assess whether the model fit could improve. However, due to the limited number of samples available in the absorption phase, the absorption-related parameters were maintained as in the original Pitsiu model. Ultimately, estimates for CL_AGE, V, IIV_CL/F, IIV_V/F, and the residual error were obtained, as presented in Supplementary Table S1. This refined model demonstrated the optimal performance with the most substantial decrease in the OFV at (−31.71). Consequently, this model was selected as the final model for the subsequent simulation step.

The GOF plot of the optimized model for cetirizine is displayed in Supplementary Figure S3, demonstrating an acceptable fit through accurate predictions of concentrations. The median difference of the estimates from the 1000-time bootstrap validation process and the final model estimate was approximately 5%. Additionally, the variation was minimal (SD ± 0.1, except for V/F), indicating stable parameter estimation. The results of the NPDE analysis are presented in Supplementary Figure S4. Both the quantile-quantile plot and the NPDE distribution histogram exhibit an approximately normal distribution, thereby suggesting that the model possesses good predictive capability.

### 3.3 Simulation of recommended dose concentrations for different age groups

**FIGURE 1:**
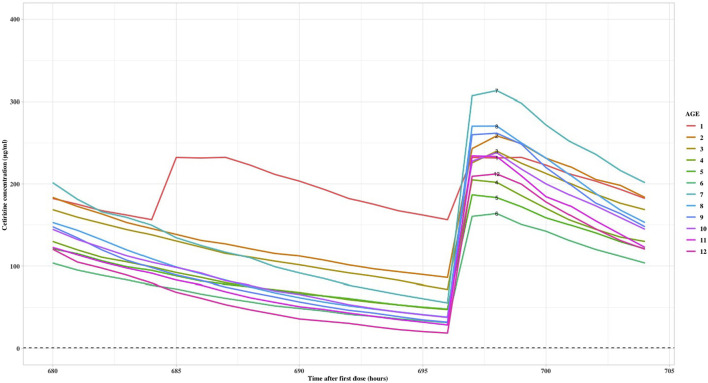
Cetirizine steady state time after first dose versus predicted concentrations with label dose.

Using the established PK model, simulated exposure over time after dosing was obtained for various age groups. In the initial simulation scenario, 12 virtual patients aged between 1 and 12 years old were generated, each receiving the maximum labeled dose. Their exposure over time-course curves for cetirizine were subsequently simulated. The PK parameters, demographic data, dosing regimens along with the steady-state peak concentrations (C_max,ss_) and trough concentrations (C_min,ss_), are summarized in Table 2. The time-course curves are depicted in Figure 1.

**TABLE 2:** Demographics and simulated results of children aged 1–12 years old given labeled doses.

Patient ID	Dose amount	Height (cm)	Age (yr)	Gender	Total body weight (kg)	Median C_max,ss_ ^a^(μg/mL)	Median C_min,ss_ ^a^(μg/mL)
1	2.5 mg bid	80	1	F	10	232.27	156.48
2	5 mg qd	88	2	F	12	243.06	86.27
3	5 mg qd	96	3	F	14	225.75	71.94
4	5 mg qd	104	4	F	17	204.98	47.53
5	5 mg qd	100	5	F	17	186.70	48.09
6	5 mg qd	120	6	F	19	160.15	31.70
7	10 mg qd	121	7	F	28	306.93	55.57
8	10 mg qd	127	8	F	33	270.42	38.28
9	10 mg qd	135	9	F	32	259.97	32.19
10	10 mg qd	146	10	F	34	227.78	37.98
11	10 mg qd	149	11	F	46	234.20	28.80
12	10 mg qd	155	12	F	40	209.28	18.94

^a^ Median prediction of 1,000 simulation results.

As evident from the table, upon administering the recommended doses of cetirizine as per its label to patients of various ages, the median C_max,ss_ ranged between 160–309 μg/mL, and the median C_min,ss_ fell within the range of 19–86 μg/mL with a once daily dosing interval (QD). Among the recommended doses, the highest C_max,ss_ was observed in the 7-year-old patient group, whereas the lowest value was observed in the 6-year-old patient group, amounting to only half of the C_max,ss_ measured in the 7-year-old group.

### 3.4 Analysis of data on overdosed pediatric patients

#### 3.4.1 Simulation of exposure in pediatric patients with doses above 0.25 mg/kg

**FIGURE 2:**
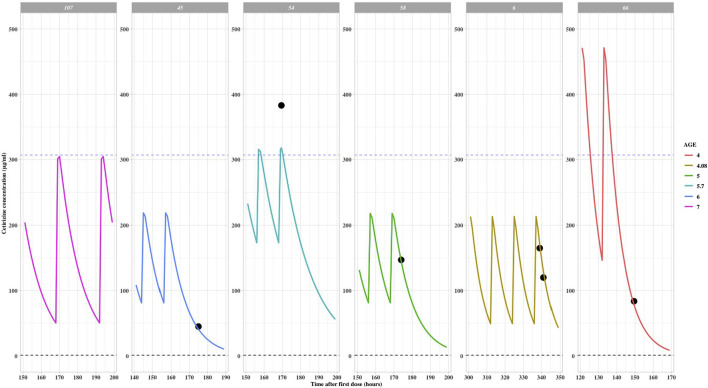
Cetirizine steady state time after first dose versus predicted concentrations with off-label dose (reference line: maximum C_max,ss_ in label dose population).

For the five pediatric patients who received doses above 0.25 mg/kg, with overdose ranges between 0.26 mg/kg to 0.45 mg/kg. The simulated C_min,ss_ and C_max,ss_ of two 4-year-old children who received higher doses (as presented in Table 2) surpassed the maximum values observed in the labeled dose group. This suggests a necessity for closely monitoring the potential risks of adverse drug reactions. The exposure curves were plotted using the actual measured data via the POSTHOC method, as depicted in Figure 2. Detailed demographic information, dosage regimens, and simulation results can be found in Table 3. All patients who received doses exceeding the recommended range were under treatment for over 1 week, or 144 h. Among the 1,000 simulation results, excluding patient 66, the median C_max,ss_ and C_min,ss_ fell within the concentration ranges specified for the labeled dose, indicative of a relatively favorable safety profile.

**TABLE 3:** Demographics and simulated results of children with off-label dose (>0.25 mg/kg).

Patient ID	Dose amount	Height (cm)	Age (yr)	Gender	Total body weight (kg)	Median C_max,ss_*(μg/mL)	Median C_min,ss_*(μg/mL)	TAFD (hr)
6	6.43 mg bid (0.38 mg/kg)	102	4.08	M	17	417.67	237.31	341
45	5 mg bid (0.26 mg/kg)	120	6	F	19	236.64	100.48	149
54	5 mg bid (0.26 mg/kg)	108	5.7	M	19	266.31	135.87	158
58	5 mg bid (0.26 mg/kg)	115	5	M	19.5	277.34	150.08	162
66	9 mg bid (0.45 mg/kg)	112	4	M	20	580.68	325.40	170

*Median prediction of 1,000 simulation results (TAFD: Time after first dose (hr)).

Patient 66 experienced an overdose due to a parental error and subsequently received continuous treatment for 1 week with a dosage greater than the recommended dose but not exceeding the recommended maximum limit. Following the final administration, the measured concentration was found to be 83.7 μg/mL, while the model-predicted concentration was 88.4 μg/mL. This indicates that the model is capable of accurately simulating the pharmacokinetic characteristics in pediatric patients. The predicted C_max,ss_ for this patient 66 was 580.68 μg/mL, and the C_min,ss_ was 325.40 μg/mL. Upon discontinuing the medication, the concentration rapidly decreased, and no adverse reactions were reported.

#### 3.4.2 Simulation and clinical applications of an overdosed pediatric patient

The established PK model was employed in a real-world setting to address a clinical overdose situation resulting from a medication error. A 5-year-old male patient had initially been prescribed a dosage regimen of 5 mg twice daily (bid). Unfortunately, due to the error, a single dose of 30 mg was administered after 12 h of administering the first dose, corresponding to 1.5 mg/kg. Upon realization of the mistake, the medication was promptly discontinued, and the patient was hospitalized for further evaluation.

**FIGURE 3:**
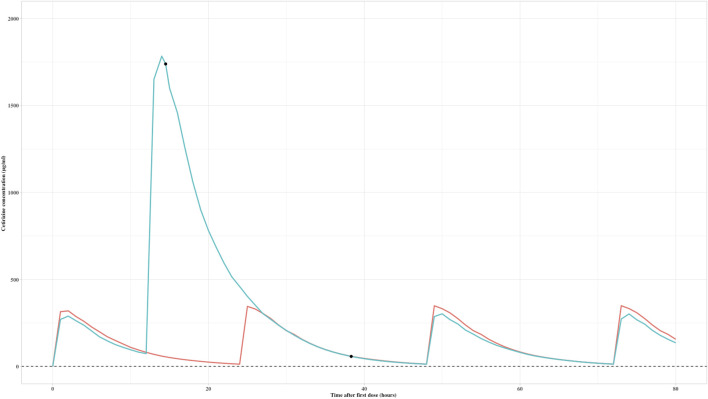
Predicted exposure over time after first dose of a child with an overdose of cetirizine (black dots are real measured values; red curve is the exposure-time curve of the child; green curve is the simulated exposure over time of 5 mg bid dose as a reference exposure).

A therapeutic drug monitoring (TDM) sample was later collected, revealing a concentration of 1725 μg/mL, taken 2.5 h after the unintended dose. Based on the simulated exposure over time result (Figure 3), it was estimated that cetirizine would be rapidly eliminated. Therefore, it was suggested that the medication could be safely restarted within 48 h of the original incorrect dose. In accordance with this advice, a subsequent TDM sample was drawn the following day, showing a concentration of 111 μg/mL, which fell within the acceptable exposure range. The patient was advised to recommence the medication starting from the third day, ensuring safe and appropriate dosing going forward.

### 3.5 Safety data analysis

The demographic characteristics of both groups did not show any statistically significant differences, as presented in Table 4. Of the 66 patients, 42 had post-treatment assessments, with data available for 22 patients who had their liver function tested after at least 3 days of treatment. These patients were presented in Table 5, Table 6 for safety analysis. No adverse reactions were noted in either group.

**TABLE 4:** Demographics and disease and treatment information in different dose groups.

Dose group	Age (yr)	Height (cm)	Weight (kg)	Gender	Dose amount (mg/day)
Regular dose (n = 39)	7.6 ± 3.9	130 ± 28	33.2 ± 17.5	Male 23	8.1 ± 3.3
				Female 16	
Exceeding dose (n = 24)	4.3 ± 1.8	105 ± 13	17.8 ± 3.4	Male 15	7.5 ± 2.3
				Female 9	
*p*-value	<0.0001	0.001	<0.0001	0.98	0.4

**TABLE 5:** Safety outcomes in different dose groups.

Dose group	ALT (U/L)	AST (U/L)	Urea (mmol/L)	Cr (μmol/L)
Regular dose (n = 31)	21.24 ± 11.82	27.64 ± 9.20	4.50 ± 1.54	46.96 ± 18.84
Exceeding dose (n = 11)	18.9 ± 8.56	33.8 ± 8.39	3.79 ± 1.16	38.55 ± 7.89
*p*-value	0.28	0.03	0.09	0.08

**TABLE 6:** Safety outcomes in different dose groups for 3 days or more of treatment.

Dose group	ALT (U/L)	AST (U/L)	Urea (mmol/L)	Cr (μmol/L)
Regular dose (n = 17)	23.91 ± 14.17	27.45 ± 7.75	4.34 ± 1.58	50.50 ± 20.78
Exceeding dose (n = 5)	21.00 ± 5.52	33.2 ± 7.79	3.68 ± 1.28	38.00 ± 7.21
*p*-value	0.33	0.09	0.22	0.04

## 4 Discussion

This study is the first to investigate the PK characteristics of cetirizine in Chinese pediatric patients. Relying on the established model, various simulation scenarios were devised with the objective of optimizing dosage for pediatric patients of differing ages. These simulations provided real-world data on drug exposure and safety, providing reference for the clinical application of double or even higher doses.

### 4.1 Model development

Due to the relatively limited sample size collected per individual in this study, particularly during the absorption phase, the published Pitsiu model was utilized for developing the Chinese pediatric model of cetirizine. Consequently, an external validation of the model was applied to the current study instead of constructing a new model based on our sparse data. To the best of our knowledge, this model is the only evidence that can be reproduced in public database. As the model already incorporates covariates, we estimated these parameters to investigate potential racial differences. All absorption-related model parameters were obtained from Pitsiu model and fixed, and specific parameters are detailed in Supplementary Table S1. The IPRED scatter plot was found to be evenly distributed on either side of the y = *x*-axis. Similarly, the CWRES scatter plot exhibited symmetry in the TAD, and the majority of the CWRES values in the final model were evenly distributed within the range of ±2, except for the largest TAD value.

Peer models, also including model of levocetirizine (Pitsiu et al., 2004; Hussein et al., 2005), have reported an underestimation of PRED and IPRED in higher concentrations where there is a high IIV of ka. Comparable baseline demographics in terms of age (with a range 0.5–12 years) and weight (with a range of 7.3–53 kg) could be observed between Pitsiu model and our dataset. Similar in Pitsiu model, age has a linear influence on CL/F and V/F, and CL/F was found to be slightly lower in female children within the same age group. In the Pitsiu model, the scaling factor of age on CL/F was 0.12, whereas it was 0.28 in our results, suggesting a more significant impact of age in the Asian population. Since no simulations were conducted in the peer-reviewed models, our simulation outcomes based on external validation can help fill the knowledge gap in clinical practice.

### 4.2 Exploration of off-label dose

In routine clinical practice, doses of cetirizine above the label recommendation are relatively common. Based on the established PPK model, exposure metrics were simulated for pediatric patients aged 1–12 years utilizing the recommended label doses. The study revealed that the C_max,ss_ for 7-year-old children was the highest (307 μg/mL), while the lowest was noted in 6-year-old children. Consequently, from a PK perspective, certain pediatric patients of specific ages may experience lower drug exposure at the recommended label dose.

In this study, five patients received doses exceeding 0.25 mg/kg for over 1 week. Despite no adverse reactions being reported, it is still advised to provide patient education and obtain informed consent to mitigate the potential risks associated with overdose.

### 4.3 Safety evaluation

This study investigated the safety of administering cetirizine at dosages higher than those recommended on the label in the Chinese pediatric population using real-world data. The findings revealed that the use of higher-dose cetirizine was indeed safe. Even in a case where a patient mistakenly received a six times higher dosage, they attained normal blood drug trough levels within a single elimination cycle, a result that was comparable to the levels seen in the group receiving the recommended dose. According to the cetirizine label, the incidence of adverse drug reactions is low and predominantly mild and transient, including symptoms such as headache, dizziness, somnolence, restlessness, dry mouth, and abdominal discomfort. However, in the present study, no such discomfort symptoms were observed among pediatric patients, nor were any abnormalities detected in liver or renal function tests. Furthermore, no adverse reactions were noted in subjects who received dosages exceeding the label-recommended amount, which underscores the favorable safety profile of the drug.

The safety data provided in this study on the use of cetirizine in Chinese pediatric patients has shown excellent tolerability and safety, regardless of whether it was administered at standard or higher doses as per the guidelines. This data offers evidence-based support for the rational utilization of cetirizine in this patient population.

### 4.4 Limitations

The primary limitation of this study was the limited sample size, which can be attributed to the challenges in conducting sampling in the pediatric population, particularly during the absorption phase when data is relatively sparse. Both the parameters of the Pitsiu model incorporating the flip-flop equation and a standard linear absorption model were tested, and a similar trend was observed in their DV (observed values) versus IPRED (individual predicted values), along with nearly identical OFVs (objective function values). Given cetirizine’s favorable safety profile, any potential bias in the absorption part would likely be insignificant. As the study’s objective was not to develop a comprehensive pharmacokinetic (PK) model but rather to perform external validation using the Pitsiu model, an accurate IPRED is sufficient for simulating trough concentrations in both labeled and off-label pediatric populations. For future research, expanding the sample size or conducting multicenter studies could enable estimation of more parameters, thereby providing a fuller understanding of the entire PK process, especially absorption.

Furthermore, due to the paucity of covariate information on body weight, weight-based simulations were not carried out to determine the optimal dosing regimens for children of differing weights within specific age ranges.

This study revealed that cetirizine demonstrated a favorable safety profile in Chinese pediatric patients when administered at off-label doses. The established PK model can be utilized for predicting drug exposure in the Chinese pediatric population, and further serves as the reference for future exposure-response analyses.

## Data Availability

The original contributions presented in the study are included in the article/Supplementary Material, further inquiries can be directed to the corresponding author.

## References

[B1] Caffarelli C. Paravati F. El Hachem M. Duse M. Bergamini M. Simeone G. (2019). Management of chronic urticaria in children: a clinical guideline. Ital. J. Pediatr. 1 (45), 101. 10.1186/s13052-019-0695-x PMC6694633 31416456

[B2] Chen C. (2008). Physicochemical, pharmacological and pharmacokinetic properties of the zwitterionic antihistamines cetirizine and levocetirizine. Curr. Med. Chem. 21 (15), 2173–2191. 10.2174/092986708785747625 18781943

[B3] Diepgen T. L. Early Treatment of the Atopic Child Study Group (2002). Long-term treatment with cetirizine of infants with atopic dermatitis: a multi-country, double-blind, randomized, placebo-controlled trial (the ETAC trial) over 18 months. Pediatr. Allergy Immunol. 4 (13), 278–286. 10.1034/j.1399-3038.2002.01047.x 12390444

[B4] FDA (2002a). Cetirizine clinical pharmacology review[EB/OL]. Available at: https://www.accessdata.fda.gov/drugsatfda_docs/nda/2002/020346_S008_ZYRTEC%20TABLETS.pdf (Accessed January 28, 2024).

[B5] FDA (2002b). Cetirizine label[EB/OL]. Available at: https://www.accessdata.fda.gov/drugsatfda_docs/label/2002/19835s15,%2020346s8lbl.pdf (Accessed January 28, 2024).

[B6] Hussein Z. Pitsiu M. Majid O. Aarons L. de Longueville M. Stockis A. (2005). Retrospective population pharmacokinetics of levocetirizine in atopic children receiving cetirizine: the ETAC study. Br. J. Clin. Pharmacol. 1 (59), 28–37. 10.1111/j.1365-2125.2005.02242.x PMC1884967 15606437

[B7] Magalhães J. Rodrigues A. T. Roque F. Figueiras A. Falcão A. Herdeiro M. T. (2015). Use of off-label and unlicenced drugs in hospitalised paediatric patients: a systematic review. Eur. J. Clin. Pharmacol. 1 (71), 1–13. 10.1007/s00228-014-1768-9 25318905

[B8] Meng M. Liu E. Zhang B. Lu Q. Zhang X. Ge B. (2022). Guideline for the management of pediatric off-label use of drugs in China (2021). BMC Pediatr. 1 (22), 442. 10.1186/s12887-022-03457-1 PMC9307429 35869466

[B9] Pitsiu M. Hussein Z. Majid O. Aarons L. de Longueville M. Stockis A. (2004). Retrospective population pharmacokinetic analysis of cetirizine in children aged 6 months to 12 years. Br. J. Clin. Pharmacol. 4 (57), 402–411. 10.1046/j.1365-2125.2003.02017.x PMC1884474 15025737

[B10] Silva D. Ansotegui I. Morais-Almeida M. (2014). Off-label prescribing for allergic diseases in children. World Allergy Organ J. 1 (7), 4. 10.1186/1939-4551-7-4 PMC3928583 24528848

[B11] Simons F. E. (1999). Prospective, long-term safety evaluation of the H1-receptor antagonist cetirizine in very young children with atopic dermatitis. ETAC Study Group. Early Treatment of the Atopic Child. J. Allergy Clin. Immunol. 2 (104), 433–440. 10.1016/s0091-6749(99)70389-1 10452767

[B12] Simons F. E. Johnston L. Simons K. J. (2000). Clinical pharmacology of the H1-receptor antagonists cetirizine and loratadine in children. Pediatr. Allergy Immunol. 2 (11), 116–119. 10.1034/j.1399-3038.2000.00045.x 10893015

[B13] Simons F. E. Silas P. Portnoy J. M. Catuogno J. Chapman D. Olufade A. O. (2003). Safety of cetirizine in infants 6 to 11 months of age: a randomized, double-blind, placebo-controlled study. J. Allergy Clin. Immunol. 6 (111), 1244–1248. 10.1067/mai.2003.1496 12789224

[B14] Spicák V. Dab I. Hulhoven R. Desager J. P. Klánová M. de Longueville M. (1997). Pharmacokinetics and pharmacodynamics of cetirizine in infants and toddlers. Clin. Pharmacol. Ther. 3 (61), 325–330. 10.1016/S0009-9236(97)90165-X 9084458

[B15] Yackey K. Stukus K. Cohen D. Kline D. Zhao S. Stanley R. (2019). Off-label medication prescribing patterns in pediatrics: an update. Hosp. Pediatr. 3 (9), 186–193. 10.1542/hpeds.2018-0168 30745323

[B16] Yang P. Zhou W. Zhang X. Zhou C. Yang L. Liu W. (2021). Determination of cetirizine in the plasma of children by HPLC - MS/MS. Chin. J. Clin. Pharmacol. 22 (37), 3161–3164. 10.13699/j.cnki.1001-6821.2021.22.035

[B17] Zhou P. Zhou W. Shen K. Hong J. Zhan S. Zhai S. (2022). Crossover replantation of a foot after bilateral traumatic lower-leg amputation. Chin. J. Evidence-based Med. 12 (22), 1–7. 10.1016/j.jpra.2022.01.005 PMC8851088 35198719

[B18] Zuberbier T. Aberer W. Asero R. Abdul Latiff A. H. Baker D. Ballmer-Weber B. (2018). The EAACI/GA^2^LEN/EDF/WAO guideline for the definition, classification, diagnosis and management of urticaria. Allergy, 7 (73), 1393–1414. 10.1111/all.13397 29336054

