# Supplementary material for: Model-based exploration of the rationality of off-label use of cetirizine in Chinese pediatric patients: a prospective cohort study
Source: Front Pharmacol. 2024 Mar 14;15:1322788. doi: 10.3389/fphar.2024.1322788 (PMC10972884; doi:10.3389/fphar.2024.1322788)
Supplement: Supplementary file 1 [file DataSheet1.docx]

**Supplements**


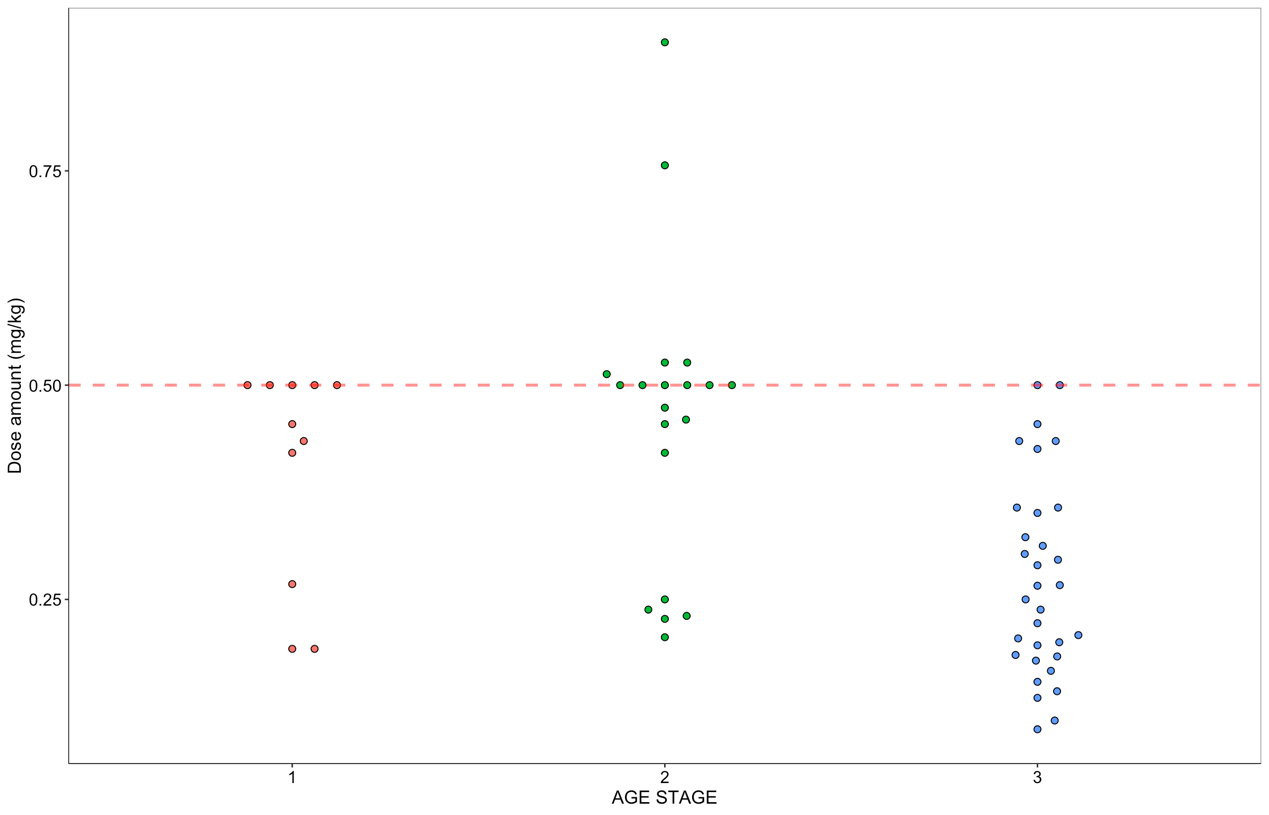


**Figure S1. Dose distribution of different ages groups according to kg body weight.**


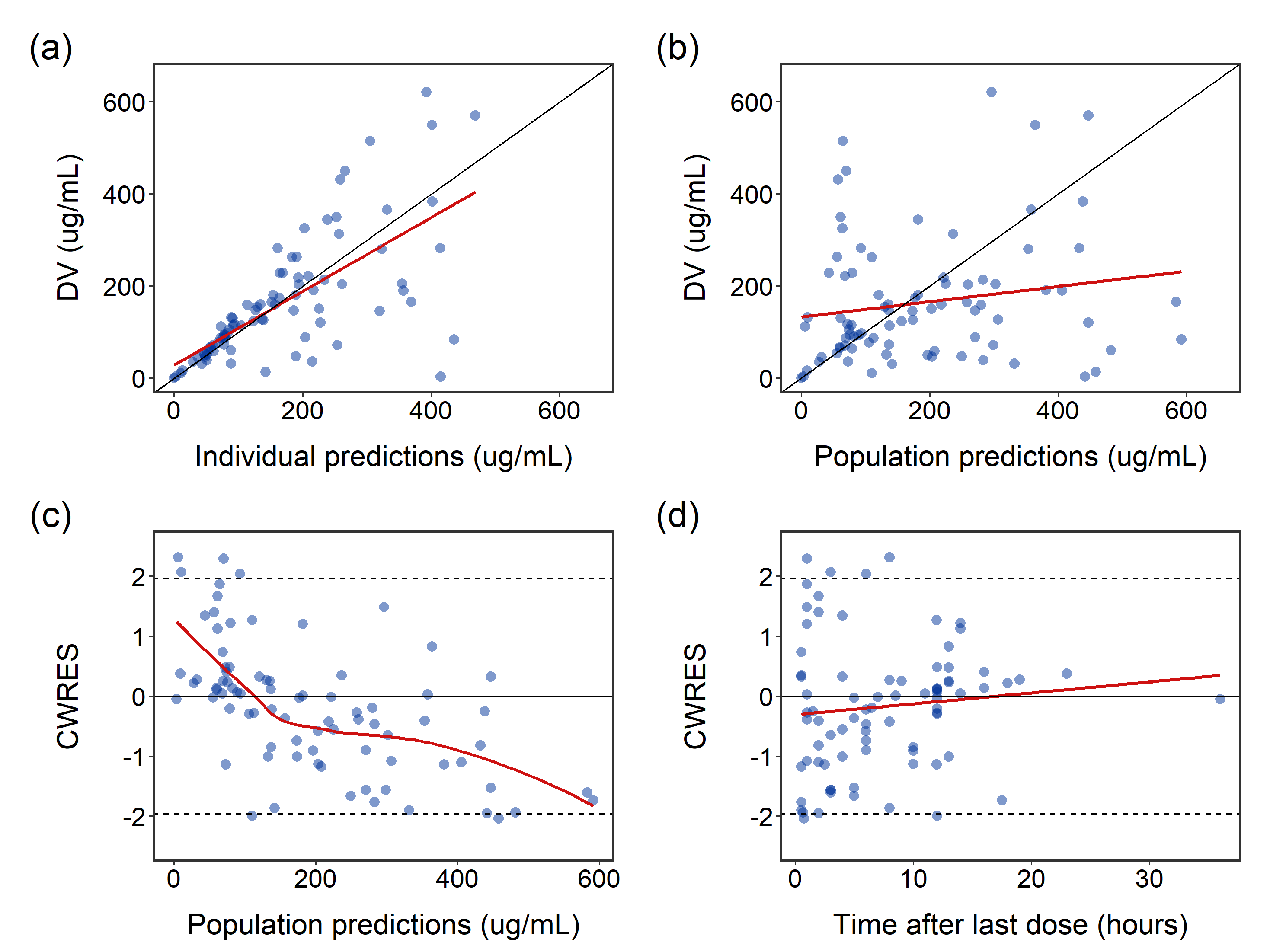


**Figure S2. GOF plot of the external evaluation of Pitsu model (POSTHOC)**


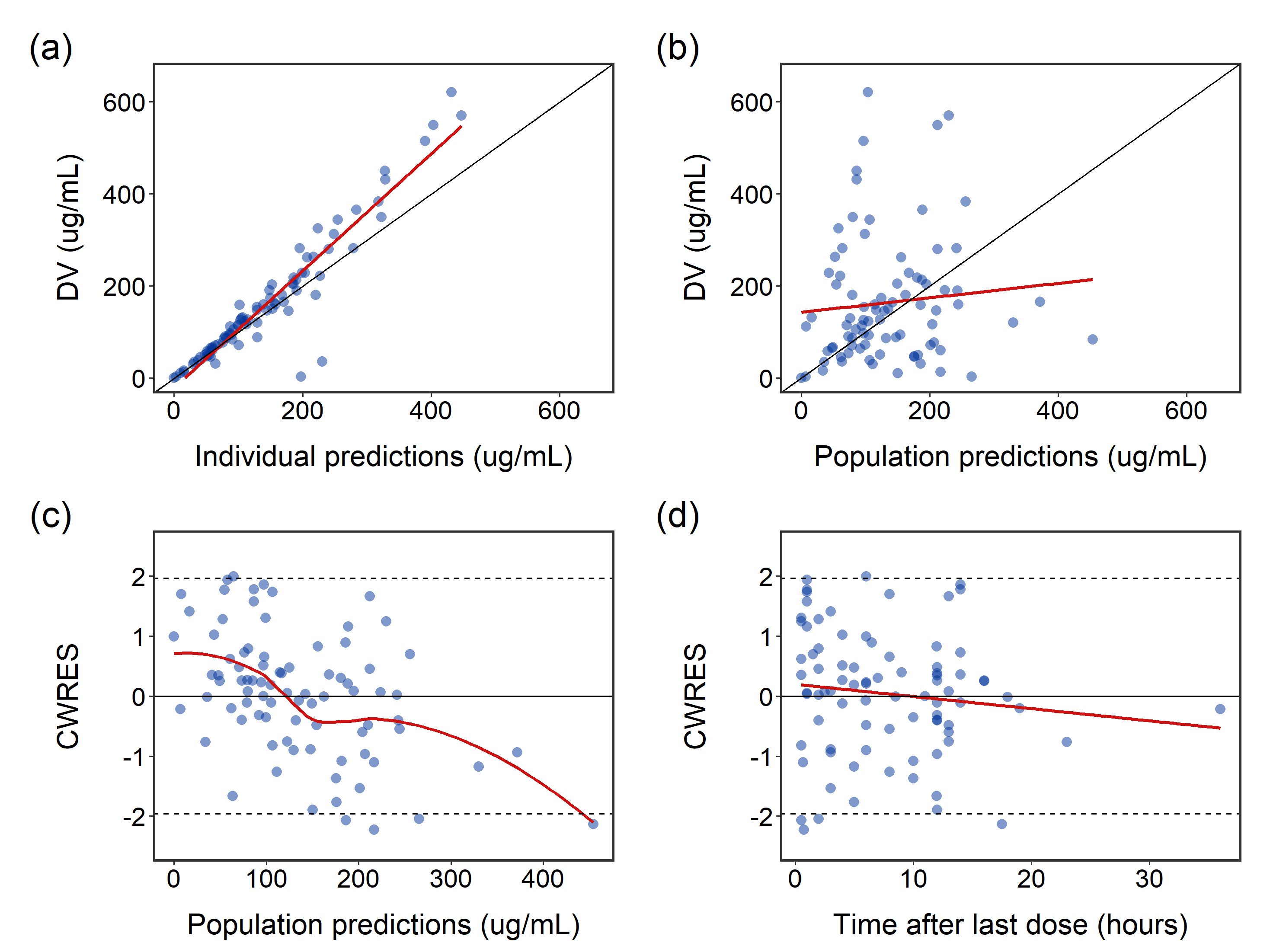


**Figure S3. GOF plots for final model evaluation**


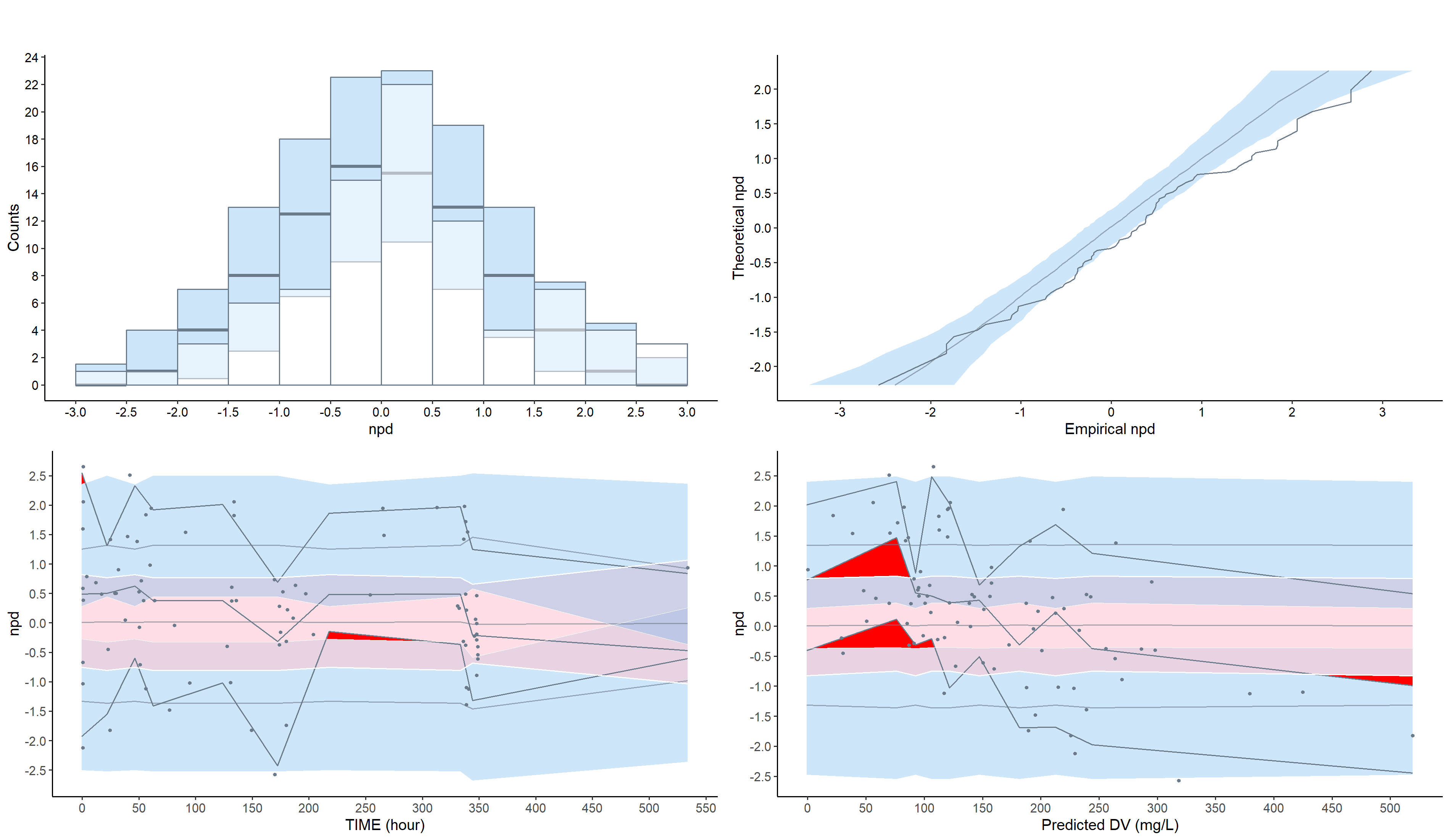


Distribution of npde :

nb of obs: 85

mean= 0.2777 (SE= 0.12 )

variance= 1.26 (SE= 0.19 )

skewness= 0.04258

kurtosis= -0.05485

---------------------------------------------

Statistical tests (adjusted p-values):

t-test : 0.0753 .

Fisher variance test : 0.324

SW test of normality : 1

Global test : 0.0753 .

---

Signif. codes: '***' 0.001 '**' 0.01 '*' 0.05 '.' 0.1

---------------------------------------------

**Figure S4. NPDE plots based on 1000 times simulation**

**Table S1. Cetirizine PK model parameter estimation**

| **Parameters** | **Typical value** | **Relative standard error (RSE) [Shrinkage%]** | **Bootstrap (Median [95%CI])** |
| --- | --- | --- | --- |
| Proportional error | 0.36 | 16% | 0.34 [0.15-0.50] |
| CL_AGE | 0.28 | 17% | 0.28 [0.20-0.38] |
| CL_SEX on Male | 0.771 FIXED | / | / |
| CL_SEX on Female | 0.592 FIXED | / | / |
| V | 23.1 | 40% | 23.45 [10.62-54.92] |
| V_AGE | 1.42 FIXED | / | / |
| Ka | 2.14 FIXED | / | / |
| IIV_CL/F | 0.378 | 34% [21.2%] | 0.35 [0.12-0.65] |
| IIV_V/F | 0.445 | 41% [47.1%] | 0.43 [0.07-1.11] |
| IIV_Ka | 2.1 FIXED | / | / |
| Sigma | 1 FIXED | / | / |
